# Effects of platelet-rich fibrin combined with hydroxyapatite in the treatment of patients with periodontal intrabony defects: a meta-analysis

**DOI:** 10.1016/j.clinsp.2026.101035

**Published:** 2026-06-29

**Authors:** Luyue Li, Tian Liu, Ziqin Yan, Yanyan Zhao

**Affiliations:** Department of Stomatology, The Central Hospital of Wuhan, Tongji Medical College, Huazhong University of Science and Technology, Hubei, China

**Keywords:** Platelet-rich fibrin, Hydroxyapatite, Intrabony defects, Meta-analysis

## Abstract

**Objective:**

The objective of this meta-analysis was to systematically assess the effectiveness of Platelet-Rich Fibrin (PRF) in combination with Hydroxyapatite (HA) for the regenerative treatment of periodontal intrabony defects

**Methods:**

A systematic search was conducted across multiple databases, including PubMed, Web of Science, Cochrane Library, EMBASE, CNKI, and Wanfang, to identify Randomized Controlled Trials (RCTs) examining the combined use of PRF and HA for periodontal intrabony defects. Two independent researchers screened the literature according to inclusion and exclusion criteria, extracted data, and assessed the quality of the studies. Data analysis was performed using Stata 17.0 software.

**Results:**

A total of 11 RCTs were included in the final meta-analysis. The meta-analysis indicated that the combination of PRF and HA significantly improved probing depth (MD = 0.74, 95% CI 0.32‒1.17, p < 0.001), clinical attachment loss (MD = 0.75, 95% CI 0.32‒1.19, p < 0.001), plaque index (MD = 0.05, 95% CI 0.00‒0.10, p = 0.03), and gingival index (MD = 0.13, 95% CI 0.01‒0.25, p = 0.04) in the treatment of periodontal intrabony defects.

**Conclusion:**

The combination of platelet-rich fibrin and hydroxyapatite appears to be an effective approach for treating periodontal intrabony defects. However, due to the limited number and quality of the included studies, further research with high-quality, large-scale trials is necessary to validate these findings.

## Highlights

• Platelet-rich fibrin plus hydroxyapatite improved probing depth.
• Platelet-rich fibrin plus hydroxyapatite improved attachment level.
• Combined therapy improved gingival and plaque index outcomes.

## Introduction

Periodontal intrabony defects are a critical manifestation of periodontitis, characterized by vertical bone loss within the alveolar bone that encircles a tooth^1^ These defects significantly impact the structural integrity and functional stability of teeth, making their effective treatment a major focus in periodontal therapy^2^ The primary objective in addressing these defects is to regenerate the lost periodontal structures, which include cementum, periodontal ligament, and alveolar bone, to restore the tooth's functionality and aesthetics^3^

Traditional treatment approaches for periodontal intrabony defects, such as scaling and root planing or surgical interventions, have yielded variable results in achieving full periodontal regeneration^4^ The unpredictable nature of these outcomes has led to the exploration of advanced regenerative techniques, including the use of biologics and biomaterials, which aim to enhance healing and promote the regeneration of periodontal tissues^5^ Platelet-Rich Fibrin (PRF) is an autologous blood-derived product that has gained popularity due to its ability to release growth factors over time, thereby promoting wound healing, angiogenesis, and tissue regeneration. PRF's ease of preparation, cost-effectiveness, and autologous nature make it a valuable tool in regenerative periodontal therapy^6^ Hydroxyapatite (HA), a biomaterial that closely resembles the mineral composition of bone, has long been used in bone grafting due to its osteoconductive properties. HA provides a scaffold that facilitates bone regeneration by supporting the attachment and proliferation of osteoblasts^7^^,^^8^

Combining PRF with HA offers a potentially synergistic approach to treating periodontal intrabony defects. While PRF enhances the biological environment for healing, HA provides structural support, promoting more effective bone regeneration. Despite promising outcomes reported in several studies, the clinical efficacy of this combination therapy remains a subject of ongoing investigation. Some studies have reported superior clinical outcomes, such as improved clinical attachment level and greater defect fill, while others suggest that the benefits are comparable to those of traditional treatments^9^, ^10^, ^11^ Given the variability in the reported outcomes, a comprehensive meta-analysis is warranted to assess the overall effectiveness of PRF combined with HA in the treatment of periodontal intrabony defects. This meta-analysis aims to systematically evaluate and synthesize the latest evidence, providing a clearer understanding of the clinical benefits and potential limitations of this combination therapy. The findings of this study will have important implications for clinical practice and future research in periodontal regeneration.

## Materials and methods

### Literature search strategy

A comprehensive search was conducted in PubMed, Web of Science, Cochrane Library, EMBASE, CNKI, and Wanfang databases to identify Randomized Controlled Trials (RCTs) examining the combined use of Platelet-Rich Fibrin (PRF) and Hydroxyapatite (HA) in the treatment of periodontal intrabony defects. The search covered publications from the inception of each database until June 2024. The search strategy employed a combination of subject terms, free terms, and Boolean operators, and reference lists of eligible studies were manually screened to ensure comprehensive coverage. The search terms used across all six databases included: “intrabony defect”, “platelet-rich fibrin”, “PRF”, and “hydroxyapatite”. The search strategy is provided in Supplementary Table 1.

### Inclusion and exclusion criteria

The inclusion criteria were as follows: 1) Participants: patients with periodontal intrabony defects, with no restrictions on age or nationality; 2) Intervention: studies were eligible if they compared PRF combined with HA ‒ with or without additional biocompatible bone substitutes ‒ against the same bone substitute material(s) used alone without PRF; 3) Outcomes: Probing Depth (PD), Clinical Attachment Loss (CAL), Plaque Index (PI), and Gingival Index (GI); 4) Study design: Randomized Controlled Trials (RCTs). The exclusion criteria included duplicate publications, studies with incomplete original data or inaccessible full-text information, reviews, case reports, systematic reviews, conference papers, animal studies, and non-clinical research.

### Data extraction and quality assessment

The retrieved literature was imported into EndNote X9 for management, and duplicates were removed. Two independent researchers screened the articles based on the inclusion criteria, with discrepancies resolved through discussion or consultation with a third party. Data extraction was performed using Excel, including details such as the first author, publication year, country, age, interventions, and follow-up duration. Quality assessment of the RCTs was conducted by two researchers using the Cochrane Collaboration's risk of bias tool, evaluating aspects such as random sequence generation, allocation concealment, blinding of participants and personnel, blinding of outcome assessment, completeness of outcome data, selective reporting, and other biases. Each item was judged as “low risk”, “high risk”, or “unclear”.

### Statistical analysis

The analysis of data was performed utilizing the Stata 17.0 software package. Relative Risk (RR) with a corresponding 95% Confidence Interval (95% CI) was employed to present categorical variables, while Standardized Mean Difference (SMD) with a 95% CI was used for continuous variables. The χ² test and *I²* statistic were applied to evaluate heterogeneity among the included studies. When *I²* was less than 50% and p-value exceeded 0.1, indicating the absence of significant heterogeneity, a fixed-effects model was implemented; otherwise, a random-effects model was adopted. Sensitivity analysis was performed using the leave-one-out method, where each study was systematically removed from the meta-analysis to evaluate the impact on pooled estimates. In cases where the number of included studies surpassed ten, a funnel plot was constructed to investigate potential publication bias.

## Results

### Literature search results and characteristics of included studies

**Fig. 1:**
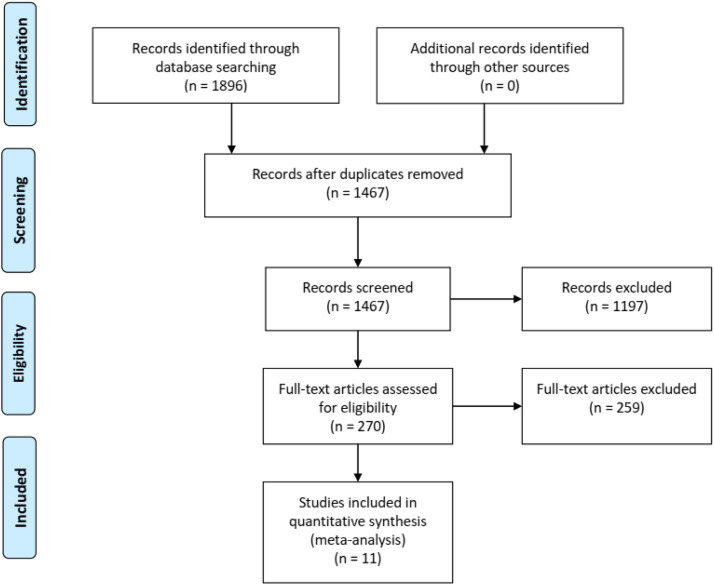
A flowchart of the meta-analysis. From: Moher D, Liberati A, Tetzlaff J, Altman DG, The PRISMA Group (2009). Preferred Reporting Items for Systematic Reviews and Meta-Analyses: The PRISMA Statement. PLoS Med 6(7):e1000097. doi:10.1371/journal.pmed1000097. For more information, visit www.prisma-statement.org.

**Fig. 2:**
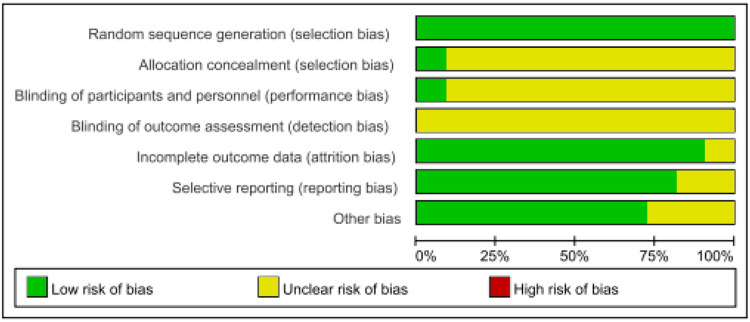
The risk of bias graph.

Fig. 1 illustrates the selection process. The database search yielded 1896 articles, of which 429 duplicates were removed. Based on title and abstract screening, 1097 articles were excluded for not meeting the study criteria. Full-text review of the remaining 270 articles resulted in the inclusion of 11 RCTs^9^, ^10^, ^11^, ^12^, ^13^, ^14^, ^15^, ^16^, ^17^, ^18^, ^19^ The basic characteristics of the included studies are presented in Table 1, and the quality assessment results are shown in Fig. 2.

**Table 1:** Characteristics of the included studies.

Study	Year	Country	Sample size	Age (years)		Treatments		Follow-up Period (months)
				Intervention	Control	Intervention	Control	
Elgendy	2015	Egypt	20	44.25±8.45	39.70±6.36	PRF±HA	HA	6
Dambhare	2019	Saudi Arabia	24	40±4.29		PRF±HA	HA	12
Chaudhary	2023	India	34	30.41±6.35	29.76±5.29	PRF±HA	HA	9
Bahammam	2021	Saudi Arabia	30	37.4 ± 4.4	40.2 ± 5.9	PRF±HA	HA	6
Baghele	2023	India	42	25‒55		PRF±HA	HA	6
Mallappa	2022	India	28	39.6 ± 4.33	40.2 ± 5.70	PRF±HA	HA	6
Qian	2014	China	20	43.7 ± 11.54	40.20±8.66	PRF±HA	HA	6
Thetay	2021	India	60	NR		PRF±HA	PRF	9
Shah	2023	India	30	31.6 ± 6.01	38.67±12.27	PRF±HA	HA	6
Pradeep	2017	India	39	39.7		PRF±HA	PRF	9
Nair	2022	India	24	33±4.2	37.5 ± 9.62	PRF±HA	HA	9

PRF, Platelet-rich fibrin; HA, Hydroxyapatite; NR, Not Reported.

### The results of the meta-analysis

**Fig. 3:**
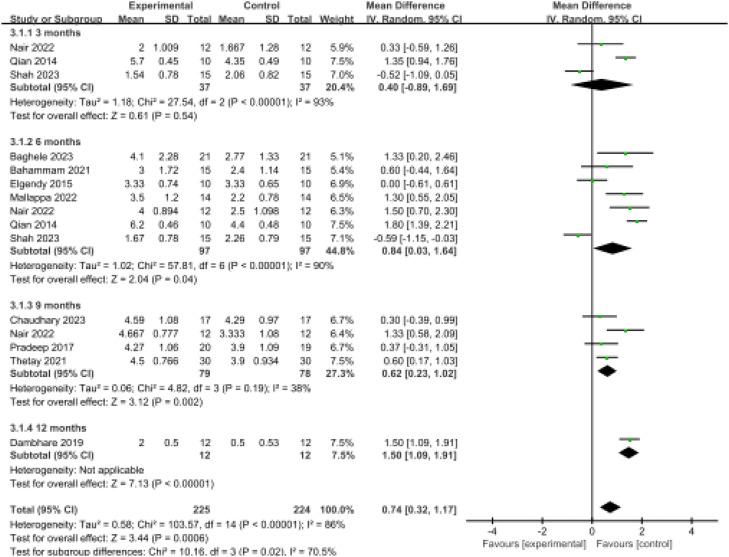
The forest plot of probing depth.

**Fig. 4:**
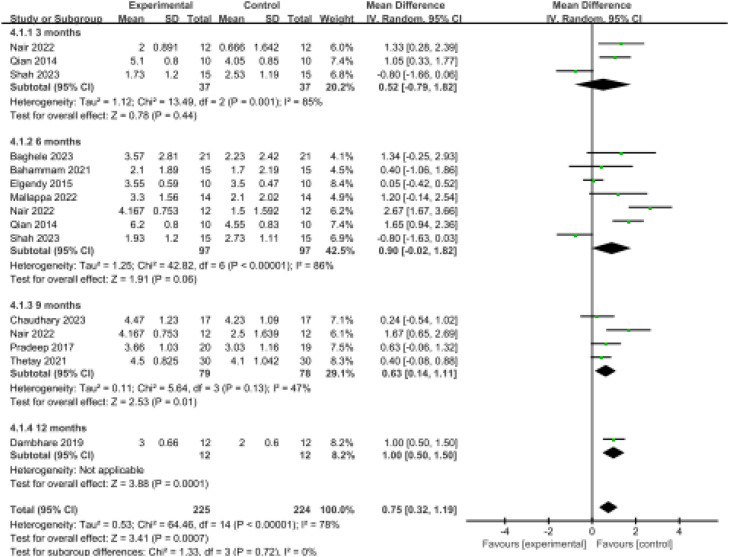
The forest plot of clinical attachment loss.

**Fig. 5:**
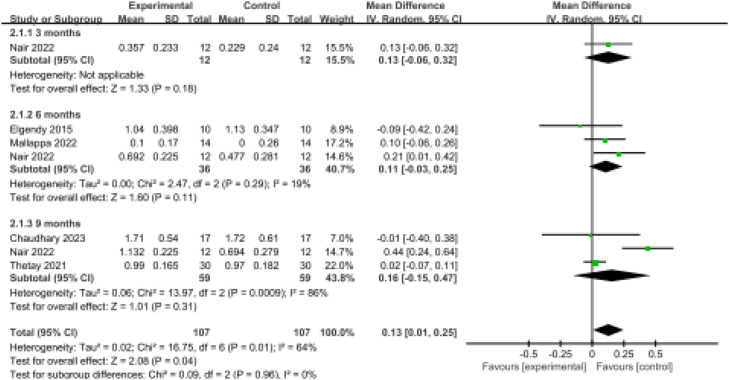
The forest plot of gingival index.

**Fig. 6:**
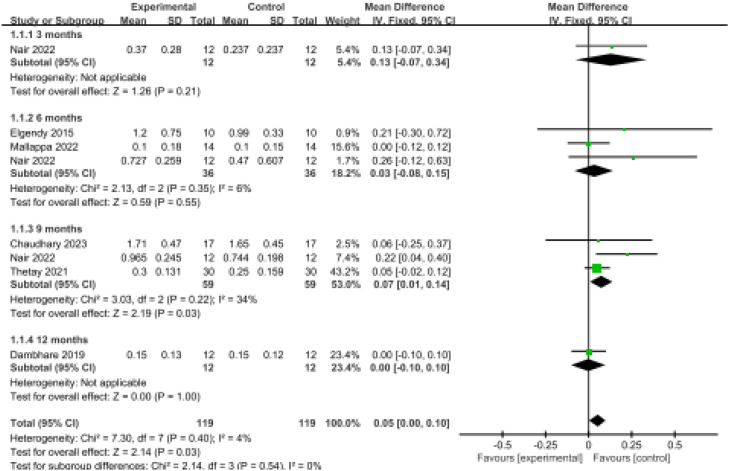
The forest plot of plaque index.

All 11 studies reported changes in PD post-treatment in the experimental and control groups. Significant heterogeneity was observed among the studies (*I²* = 86%, *p* *<* 0.001), prompting the use of a random-effects model. Meta-analysis results showed that the changes in PD at 6-, 9-, and 12-months post-surgery were (MD = 0.84, 95% CI 0.03‒1.64, *p* = 0.04), (MD = 0.62, 95% CI 0.23‒1.02, *p* = 0.002), and (MD = 1.50, 95% CI 1.09‒1.91, *p* < 0.001), respectively, with an overall significant difference (MD = 0.74, 95% CI 0.32‒1.17, *p* < 0.001) (Fig. 3). Similarly, all 11 studies reported changes in CAL post-treatment, with significant heterogeneity (*I²* = 78%, *p* < 0.001) leading to the use of a random-effects model. Meta-analysis results showed that the changes in CAL at 9- and 12-months post-surgery were (MD = 0.63, 95% CI 0.14‒1.11, *p* = 0.01) and (MD = 1.00, 95% CI 0.50‒1.50, *p* < 0.001), respectively, with an overall significant difference (MD = 0.75, 95% CI 0.32‒1.19, *p* < 0.001) (Fig. 4). Five studies reported changes in GI post-treatment. Significant heterogeneity was found (*I²* = 64%, *p* = 0.01), leading to the use of a random-effects model. Meta-analysis results indicated that changes in GI at 3-, 6-, and 9-months post-surgery were not statistically significant (MD = 0.13, 95% CI −0.06‒0.32, *p* = 0.18), (MD = 0.11, 95% CI −0.03‒0.25, *p* = 0.11), and (MD = 0.16, 95% CI −0.15‒0.47, *p* = 0.31), respectively. However, overall analysis showed a statistically significant difference (MD = 0.13, 95% CI 0.01‒0.25, *p* = 0.04), indicating a significant difference between the experimental and control groups (Fig. 5). Six studies reported changes in PI post-treatment, with no significant heterogeneity (*I²* = 4%, *p* = 0.40), prompting the use of a fixed-effects model. Meta-analysis results showed that changes in PI at 3-, 6-, and 12-months post-surgery were not statistically significant (MD = 0.13, 95% CI −0.07–0.34, *p* = 0.21), (MD = 0.03, 95% CI −0.08‒0.15, *p* = 0.55), and (MD = 0.00, 95% CI −0.10‒0.10, *p* = 1.00), respectively, while the change at 9-months post-surgery was statistically significant (MD = 0.07, 95% CI 0.01‒0.14, *p* = 0.03). Overall analysis indicated a statistically significant difference (MD = 0.05, 95% CI 0.00‒0.10, *p* = 0.03), suggesting a significant difference between the experimental and control groups (Fig. 6).

### Sensitivity analysis

Conducting a sensitivity analysis by omitting individual studies in sequence revealed that the study's conclusions were reliable.

### Publication bias

**Fig. 7:**
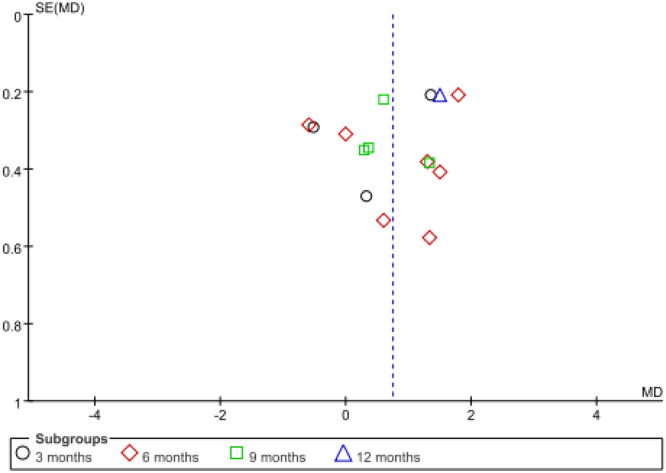
The funnel plot of this study.

The funnel plot showed that the points were symmetrically distributed on both sides of the triangle, indicating no significant risk of publication bias and that the study results are reliable (Fig. 7).

## Discussion

The present meta-analysis aimed to systematically evaluate the efficacy of PRF in combination with HA for the regenerative treatment of periodontal intrabony defects. The pooled results from 11 RCTs demonstrated that PRF+HA yielded superior clinical outcomes compared to control treatments in terms of PD reduction, CAL gain, and improvements in GI and PI at 6- to 12-month post-treatment. The overall effect estimates favored the PRF+HA group, with statistically significant differences.

The present findings are consistent with recent systematic reviews and meta-analyses evaluating the use of PRF alone or as an adjunct to bone grafting materials for periodontal intrabony defects. Miron et al^20^ reported significant improvements in PD and CAL with PRF compared to open flap debridement, while Ye et al^21^ found that PRF, alone or combined with other biomaterials, provided superior outcomes to conventional surgical treatments. The congruence in results across studies supports the clinical benefits of incorporating PRF into regenerative protocols. Notably, the meta-analysis specifically focused on the combination of PRF with HA, a widely used alloplastic bone substitute. The additive effects observed in the pooled estimates highlight the synergistic potential of coupling an autologous blood-derived scaffold like PRF with an osteoconductive material like HA.

The regenerative capacity of PRF stems from its ability to serve as a reservoir of growth factors, cytokines, and platelets that are essential for wound healing and tissue regeneration^22^ The fibrin matrix of PRF acts as a bioactive scaffold that facilitates cell migration, proliferation, and differentiation while providing mechanical stability to the defect site^23^ HA, on the other hand, closely resembles the mineral component of bone and has been shown to promote osteoblastic differentiation and new bone formation^24^ When combined, PRF and HA create a favorable microenvironment for the coordinated regeneration of periodontal tissues, including cementum, periodontal ligament, and alveolar bone^25^ Preclinical studies have elucidated the cellular and molecular mechanisms underlying the regenerative effects of PRF+HA, such as enhanced osteoblast adhesion and mineralization, upregulation of osteogenic markers, and increased vascularization^26^^,^^27^

The synergistic effects of PRF and HA can be explained through complementary biological mechanisms. PRF serves as a natural reservoir of growth factors, including PDGF, TGF-β, and VEGF, which are released gradually over 7‒14 days,^28^^,^^29^ promoting angiogenesis and cellular proliferation. The fibrin matrix provides immediate hemostasis and serves as a scaffold for cell migration^30^ HA, with its osteoconductive properties, provides structural stability and creates a favorable microenvironment for osteoblast attachment and mineralization^31^^,^^32^ Recent in vitro studies demonstrate that the combination of PRF with HA and other bone substitute materials significantly enhances cellular responses by improving osteoblast viability, migration, proliferation, and differentiation markers, including alkaline phosphatase, collagen-1, and BMP-2 expression^33^^,^^34^ Additionally, PRF increases osteoblast attachment, proliferation, and collagen-related protein production,^35^ while nanocrystalline hydroxyapatite's increased surface area enhances protein adsorption and osteoblast adhesion^36^ The combination creates a biphasic regenerative environment: immediate biological activation through PRF and sustained structural support through HA.

The positive results of this meta-analysis have important implications for clinical decision-making in the management of periodontal intrabony defects. PRF+HA may be considered a viable treatment option for cases with deep, non-contained defects that require both space maintenance and biologic mediators for optimal regeneration^37^ The technique is relatively straightforward, involving the preparation of an autologous PRF membrane or plugs to be mixed with HA granules and grafted into the defect^38^ Compared to other regenerative approaches, such as barrier membranes or enamel matrix derivatives, PRF+HA offers advantages in terms of cost-effectiveness, ease of use, and elimination of the need for a second surgical site^39^ However, proper case selection, flap design, and surgical management are critical to achieving predictable outcomes^40^

Based on our findings, the PRF+HA combination is particularly indicated for: 1) Deep intrabony defects (≥ 5 mm) with adequate keratinized tissue. 2) Two-wall and three-wall defects with good blood supply. 3) Non-smoking patients with good oral hygiene compliance. Operative considerations include: 1) PRF preparation should follow standardized protocols (400 *g* × 10 min centrifugation). 2) HA particle size of 250‒500 μm appears optimal based on included studies. 3) Primary closure is essential for predictable outcomes. 4) Minimum 6-month follow-up is recommended for full regenerative assessment.

Several important limitations should be acknowledged in this meta-analysis. First, geographic and selection biases may limit the generalizability of findings, as over half of the included studies were conducted in India, and the literature search was restricted to Chinese and English publications. Second, substantial methodological heterogeneity was observed across studies, particularly regarding PRF preparation protocols (varying centrifugation parameters, blood collection volumes, and activation methods) and the inclusion of injectable PRF formulations without separate analysis. This heterogeneity likely contributed to the significant statistical heterogeneity observed in primary outcomes (*I²* > 50%) and may compromise the reliability of pooled estimates. Third, the meta-analysis was constrained by the limited number of included studies and small sample sizes within individual trials, which precluded comprehensive exploration of heterogeneity sources and identification of optimal treatment protocols. Fourth, follow-up data at extended time points (9- and 12-months) were derived from a limited number of studies, potentially affecting the robustness of long-term outcome assessments. Finally, standardized radiographic bone fill assessment was lacking across studies, with only three studies reporting radiographic outcomes using varied measurement methodologies, preventing meaningful quantitative synthesis of objective bone regeneration parameters. Future studies should prioritize the inclusion of studies with standardized PRF preparation protocols, comprehensive reporting of treatment variables (including HA particle characteristics and defect morphology), and uniform radiographic assessment methods to enable more robust meta-analyses and clinical recommendations.

## Conclusions

In conclusion, the present meta-analysis provides evidence supporting the use of PRF in combination with HA for the regenerative treatment of periodontal intrabony defects. The superior improvements in clinical parameters observed with PRF+HA compared to control treatments underscore its potential as a predictable and cost-effective approach for promoting periodontal regeneration. The findings of this study contribute to the growing body of literature on PRF-based therapies and offer valuable insights to guide clinical practice. However, further high-quality randomized controlled trials are warranted to confirm the long-term effectiveness of PRF+HA and elucidate the factors influencing treatment outcomes. Continued research efforts in this area will advance our understanding of periodontal wound healing and lead to the development of optimized regenerative strategies that can effectively restore the health and function of compromised tooth-supporting tissues.

## Data availability

The datasets used and/or analyzed during the current study are available from the corresponding author on reasonable request.

## Ethical statement

As this is a secondary literature-based study, ethical approval is not necessary.

## Authors’ contributions

Luyue Li: Conceptualization, Data curation, Formal analysis, Investigation, Writing - original draft, Writing - review & editing. Tian Liu: Conceptualization, Data curation, Formal analysis, Investigation, Writing - original draft, Writing - review & editing. Ziqin Yan: Writing - original draft. Yanyan Zhao: Conceptualization, Methodology, Supervision, Project administration, Writing - review & editing.

## Funding

No funding was received for this work.

## Declaration of competing interest

The authors declare no conflicts of interest.

## Appendix Supplementary materials

Image, application 1
