# Supplementary material for: Effects of platelet-rich fibrin combined with hydroxyapatite in the treatment of patients with periodontal intrabony defects: a meta-analysis
Source: Clinics (Sao Paulo). 2026 Jun 29;81:101035. doi: 10.1016/j.clinsp.2026.101035 (PMC13330507; doi:10.1016/j.clinsp.2026.101035)
Supplement: Supplementary file 1 [file mmc1.docx]

**CLINICS-D-25-00514_ Supplementary Material**

**Supplementary Table 1** The search strategy for each database.

| **PubMed Search Strategy:** |
| --- |
| **#1** "periodontal diseases"[MeSH Terms] OR "alveolar bone loss"[MeSH Terms] OR "periodontal pocket"[MeSH Terms] OR "intrabony defect*"[Title/Abstract] OR "intra-bony defect*"[Title/Abstract] OR "intraosseous defect*"[Title/Abstract] OR "angular defect*"[Title/Abstract] |
| **#2** "Platelet-Rich Fibrin"[MeSH Terms] OR "platelet rich fibrin"[Title/Abstract] OR "PRF"[Title/Abstract] OR "platelet-derived fibrin"[Title/Abstract] OR "leucocyte platelet rich fibrin"[Title/Abstract] OR "L-PRF"[Title/Abstract] |
| **#3** "Durapatite"[MeSH Terms] OR "Hydroxyapatite"[Title/Abstract] OR "hydroxylapatite"[Title/Abstract] OR "HA"[Title/Abstract] OR "calcium phosphate"[Title/Abstract] |
| **#4** "randomized controlled trial"[Publication Type] OR "randomized"[Title/Abstract] OR "randomly"[Title/Abstract] |
| #1 AND #2 AND #3 AND #4 |
|  |
| **Web of Science Search Strategy:** |
| TS=(("intrabony defect*" OR "intra-bony defect*" OR "intraosseous defect*" OR "angular defect*" OR "periodontal defect*") AND ("platelet rich fibrin" OR "PRF" OR "L-PRF" OR "leucocyte platelet rich fibrin") AND ("hydroxyapatite" OR "hydroxylapatite" OR "HA" OR "calcium phosphate")) AND DT=(article) AND LA=(English OR Chinese) |
|  |
| **Cochrane Library Search Strategy:** |
| #1 MeSH descriptor: [Alveolar Bone Loss] explode all trees |
| #2 (intrabony defect* OR intraosseous defect*):ti,ab,kw |
| #3 #1 OR #2 |
| #4 MeSH descriptor: [Platelet-Rich Fibrin] explode all trees |
| #5 (platelet rich fibrin OR PRF OR L-PRF):ti,ab,kw |
| #6 #4 OR #5 |
| #7 (hydroxyapatite OR hydroxylapatite OR HA):ti,ab,kw |
| #8 #3 AND #6 AND #7 |
|  |
| **EMBASE Search Strategy:** |
| ('intrabony defect'/exp OR 'intrabony defect*':ti,ab OR 'intraosseous defect*':ti,ab OR 'angular defect*':ti,ab) AND ('platelet rich fibrin'/exp OR 'platelet rich fibrin':ti,ab OR 'prf':ti,ab OR 'l-prf':ti,ab) AND ('hydroxyapatite'/exp OR 'hydroxyapatite':ti,ab OR 'ha':ti,ab) AND ('randomized controlled trial'/exp OR 'randomized':ti,ab OR 'randomly':ti,ab) |
|  |
| **Chinese Database Search Strategy (CNKI & Wanfang):** |
| ("骨内袋" OR "牙周骨内袋" OR "角形骨缺损" OR "骨下袋") AND ("富血小板纤维蛋白" OR "PRF") AND ("羟基磷灰石" OR "HA") |
